# Intraoperative assessment of reduction and implant placement in acetabular fractures—limitations of 3D-imaging compared to computed tomography

**DOI:** 10.1186/s13018-018-0780-7

**Published:** 2018-04-10

**Authors:** Holger Keil, Nils Beisemann, Marc Schnetzke, Sven Yves Vetter, Benedict Swartman, Paul Alfred Grützner, Jochen Franke

**Affiliations:** Clinic for Trauma and Orthopaedic Surgery, BG Trauma Center Ludwigshafen at the University of Heidelberg, MINTOS – Medical Imaging and Navigation in Trauma and Orthopaedic Surgery, Ludwig-Guttmann-Str. 13, 67071 Ludwigshafen, Germany

**Keywords:** Intraoperative 3D imaging, Acetabular fracture, Computed tomography, Trauma

## Abstract

**Background:**

In acetabular fractures, the assessment of reduction and implant placement has limitations in conventional 2D intraoperative imaging. 3D imaging offers the opportunity to acquire CT-like images and thus to improve the results. However, clinical experience shows that even 3D imaging has limitations, especially regarding artifacts when implants are placed. The purpose of this study was to assess the difference between intraoperative 3D imaging and postoperative CT regarding reduction and implant placement.

**Methods:**

Twenty consecutive cases of acetabular fractures were selected with a complete set of intraoperative 3D imaging and postoperative CT data. The largest detectable step and the largest detectable gap were measured in all three standard planes. These values were compared between the 3D data sets and CT data sets. Additionally, possible correlations between the possible confounders age and BMI and the difference between 3D and CT values were tested.

**Results:**

The mean difference of largest visible step between the 3D imaging and CT scan was 2.0 ± 1.8 mm (0.0–5.8, *p* = 0.02) in the axial, 1.3 ± 1.4 mm (0.0–3.7, *p* = 0.15) in the sagittal and 1.9 ± 2.4 mm (0.0–7.4, *p* = 0.22) in the coronal views. The mean difference of largest visible gap between the 3D imaging and CT scan was 3.1 ± 3.6 mm (0.0–14.1, *p* = 0.03) in the axial, 4.6 ± 2.7 mm (1.2–8.7, *p* = 0.001) in the sagittal and 3.5 ± 4.0 mm (0.0–15.4, *p* = 0.06) in the coronal views. A positive correlation between the age and the difference in gap measurements in the sagittal view was shown (rho = 0.556, *p* = 0.011).

**Conclusions:**

Intraoperative 3D imaging is a valuable adjunct in assessing reduction and implant placement in acetabular fractures but has limitations due to artifacts caused by implant material. This can lead to missed malreduction and impairment of clinical outcome, so postoperative CT should be considered in these cases.

## Background

The acetabulum is the pelvic part of the hip joint, being formed by the ischium, ilium, and the pubis. It is a central part of the musculoskeletal system and needs to bear heavy forces due to the large range of motion of the hip joint and weight supply during walking and running. Thus, the microstructure of the acetabulum provides a very stable counterpart to the femoral head. Biomechanically, there are two bony regions that transfer the load towards the pelvic ring and the spine, referred to as anterior and posterior column.

Fractures of the acetabulum are rather rare fractures with an incidence of about 5 per 100,000 people per year, especially when compared to the incidence of proximal femur fractures that show an up to hundred-fold higher incidence, depending on the geographical region [1]. Like other fractures, acetabular fractures show a bimodal distribution. There is a peak in young, rather male patients due to high-energy traumata, for example, due to vehicle collisions or fall from height and a second peak in elder, rather female patients due to low-energy traumata combined with decreased bone density.

Due to the complex anatomy with soft tissue coverage, strong muscle tear on the inner and outer surface of the acetabulum and a concave joint surface, the assessment of the articular joint surface with intraoperative 2D imaging is limited. The crucial aspect is the weight-bearing zone of the acetabular roof where steps and gaps need to be reduced as far as possible.

Depending on the fracture type, typical surgical approaches include the Kocher-Langenbeck approach to the posterior column, and the Stoppa- as well as the ilio-inguinal approach to the anterior column. Usually, the osteosynthesis is done with 3.5 mm plates combined with single lag screws, if necessary. To increase mechanical stability of the fitting, it may be helpful to position screws close to the acetabular surface. Due to the abovementioned aspects of the anatomical situation, assessing these screws in 2D imaging is extremely challenging and it is not always possible to precisely exclude extra-articular positioning [2].

As in acetabular fractures, there are several other anatomical regions that can only be assessed very limited in 2D imaging due to concave joint surfaces and anatomical configuration. This includes fractures of the tibial head as well as the calcaneus, ankle injuries involving the syndesmosis, and spinal injuries [3–6]. To improve the surgeon’s possibilities to assess the result of the reduction and osteosynthesis intraoperatively, intraoperative 3D imaging with a mobile C-arm has been available since 2001. These devices create computed tomography (CT-) like datasets by motorized movement of the C-arm around the patient and automatized acquisition of images. These images are converted to 3D volumes by methods comparable to CT-reconstruction and can be assessed directly by the surgeon. With the help of this technique, it is possible to intraoperatively exclude malreduction of the fracture and misplacement of implants. Usually, the 3D scan is performed when reduction and implant placement are considered correct in fluoroscopy. Several studies show intraoperative revision rates depending on the anatomical region of up to 40% as a consequence of the 3D imaging [7–10]. Due to these results, 3D imaging has become very common in the operative treatment of complex articular fractures.

In our institution, a 3D scan is performed in every acetabular fixation when reduction and implant placement is considered to be correct. Usually, a postoperative CT scan is not necessary as reduction and implant placement are evaluated intraoperatively. There are special circumstances, however, when a postoperative CT scan is performed. This includes two-column fractures with a two-stage approach. In these cases, usually a CT scan is performed for evaluating the whole pelvic anatomy after the first stage to be able to anticipate the second stage. Other indications include control scans of retroperitoneal hematomas or diagnostic scans for abdominal complains. In these scans, sometimes discrepancies to the intraoperative 3D scans were noticed regarding remaining steps and gaps that were not visible in the 3D scans, especially due to artifacts resulting from the implants.

The aim of this retrospective study was to evaluate the image quality and assessability of intraoperative 3D scans compared to postoperative CT scans regarding visible steps and gaps in the articular surface of the acetabulum.

## Methods

Anonymized image sets from patients that were operated on the acetabulum and received an intraoperative 3D as well as a postoperative CT scan before ambulation was included in this study.

The intraoperative scans were performed using the 3D C-arm Arcadis Orbic (Siemens, Erlangen, Germany). The postoperative CT scans were performed by the radiological department with a clinical 32-slice CT scanner (Aquilion 32, Toshiba, Japan).

Multiplanar reconstructions of the 3D data were performed to match the standard reconstruction views of the CT scans done by the radiological department (orthogonal views in standard axial, coronal, and sagittal orientation). To achieve comparable conditions for assessing the 3D data as well as the CT data, slice reconstruction thickness was set to 2 mm in all MPRs of as well 3D as CT data.

All images were assessed regarding the largest visible step and largest visible gap in each plane. “Largest” was defined as the measurement in the slice where the distinct distance between the two ends of the step, respectively gap, as described below was clearly identifiable. This was done with the use of high-contrast displays with free arrangement of contrast, brightness, and zoom by the examiner using the standard clinical PACS viewer (Impax, Agfa, Belgium). All images were assessed by two members of the research group (MS, who is an experienced consultant and NB, who is a resident), and the average values of the measurements were used for further analysis.

**Fig. 1:**
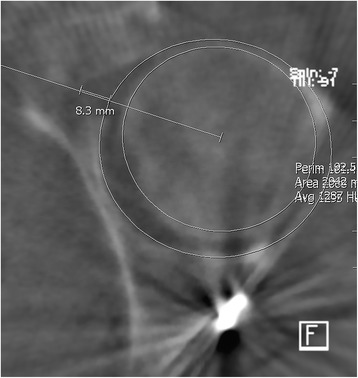
Example of intraoperative 3D scan (axial plane) with measurement lines for steps

Steps were measured in relation to the remaining articular surface. A line through the center of the femoral head was used to measure the exact distance (see Fig. 1). This was done for the largest step that was clearly identifiable in the current slice. The procedure was repeated for all three planes.

**Fig. 2:**
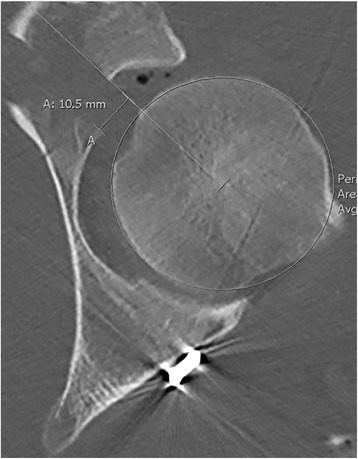
Example of postoperative CT scan (axial plane) with measurement lines for gaps

Gaps were measured relating to the virtual continuation of the articular surface. A line through the center of the femoral head was used as a reference for the distance of the distal fragment to the remaining articular surface. The least distance was used as the value for the gap (see Fig. 2).

In cases with minor or major limitations of the assessability, meaning relevant parts of the acetabular surface were overlapped by artifacts, only distinct steps and gaps were measured. Following this, if relevant steps or gaps were overlapped, they were not covered by the measurement and thus leading to underestimation of the values.

To illustrate the impact of underestimation, relative differences were calculated. For statistical analysis, the absolute value of the difference between the values measured in the 3D scans and those measured in the CT scans was used to avoid value balancing by relative differences.

The image data of the 3D scans were assessed regarding the visibility of the articular surface of the acetabulum and the femoral head according to the score shown in Table 1:

**Table 1:** Assessability score for the 3D scan data

	Yes	No
Cortical bone of the femoral head is visible in all slices	1	0
Subchondral bone of the acetabulum is visible in more than 2/3 of all slices	1	0
Points	Result	
0	Major limitations	
1	Minor limitations	
2	No limitations	

All radiological data were assessed in randomized order independently by two physicians.

Additionally, the following demographic data was collected from the patient records:

- Age
- Mechanism of accident
- Body mass index (BMI)
- Days to surgery
- Injury type (AO classification)

The following perioperative data was also analyzed:

- Duration of surgery
- Applied blood products (packed red blood cells, RBC and fresh frozen plasma, FFP)
- Number of intraoperative 3D scans (in case of multiple scans, only the last one was used for this study)
- Intraoperative revision due to findings in the 3D scan

Age was considered as a potential parameter for decreased bone density (and thus decreased contrast in X-ray examinations) and was tested for correlation to the mean difference values using Spearman’s rho.

The BMI was considered as a parameter for the amount of soft tissue surrounding the acetabulum and thus confine image quality in both 2D and 3D images and tested for correlation to the mean difference values using Spearman’s rho.

Statistical analysis was done with SPSS Version 23 (IBM, USA). Comparison of mean values was performed with Student’s *t* test. Correlation was tested with Spearman’s rho. Statistical significance was assumed when *p* < 0.05.

## Results

Twenty consecutive image sets of patients (9 males and 11 females) that were operated between August 2012 and May 2016 were included in this study. The mean age was 44.0 ± 18.6 [15.4–82.1] years. In 11 cases, the mechanism of the accident was a high-energy vehicle crash, in 3 cases a bicycle crash and in 6 cases a fall from height.

The mean BMI was 24.5 ± 4.3 [19.2–36.7] kg/m^2^, the average time between the accident and surgery was 7.9 ± 4.5 [0–19] days.

The distribution of fracture types according to the AO classification is shown in Table 2.

**Table 2:** Distribution of the fracture types according to the AO classification

Fracture type	A1	A2	A3	B1	B2	B3	C1	C2	C3
Frequency	1	1	2	1	0	3	1	9	2

Six patients (30%) were operated with a two-step approach, and 14 patients (70%) were operated with a one-step approach. In 13 cases (65%), additional cannulated screws were placed in the acetabular dome. The mean duration of surgery was 250.0 ± 103.7 [71–411] min. A mean of 1.7 ± 1.7 [0–5] units of packed red blood cells and 0.4 ± 0.9 [0–3] units of fresh frozen plasma were dispensed.

There were no serious peri- or postoperative complications like major bleeding, circulatory depression, neurological, or urological deficits.

In four cases (20%), an intraoperative correction of the reduction or implant placement was done as a result of the 3D imaging. In all cases, an additional 3D scan was performed after the correction to ensure the resulting reduction and implant position.

The assessability score (see Table 1) was 0 (major limitations) in 2 cases (10%), 1 (minor limitations) in 9 cases (45%) and 2 (no limitations) in 9 cases (45%).

The mean absolute values for the differences between the measurements in the 3D scan and the CT scans are shown in Tables 3 and 4. As shown, there was statistical significance regarding the values of visible steps in the axial views (*p* = 0.024) as well as the values of visible gaps in the axial (*p* = 0.030) and sagittal (*p* = 0.001) views. As the absolute values of the difference in the measurements were used, the absolute difference is not the difference between the displayed mean values for the modalities.

**Table 3:** Mean values of the largest visible step in the 3D and CT scans

Steps [mm]	Axial	*p* value	Sagittal	*p* value	Coronal	*p* value
3D scan	1.14 ± 1.81		1.06 ± 1.49		1.68 ± 1.46	
CT scan	2.87 ± 2.69		1.95 ± 2.18		3.14 ± 3.14	
Relative difference (3D-CT)	−1.74 ± 2.00		−0.89 ± 1.71		−1.46 ± 2.71	
Absolute difference	1.96 ± 1.78	0.024*	1.32 ± 1.38	0.149	1.88 ± 2.42	0.221

Values marked with an asterisk (*) show statistical significance

**Table 4:** Mean values of the largest visible gap in the 3D and CT scans

Gaps [mm]	Axial	*p* value	Sagittal	*p* value	Coronal	*p* value
3D scan	3.42 ± 3.32		2.02 ± 3.89		2.45 ± 1.81	
CT scan	6.41 ± 4.71		5.87 ± 3.61		4.91 ± 4.67	
Relative difference (3D-CT)	−2.99 ± 3.75		−3.85 ± 3.73		−2.46 ± 4.71	
Absolute difference	3.14 ± 3.61	0.030*	4.57 ± 2.74	0.001*	3.50 ± 3.96	0.060

Values marked with an asterisk (*) show statistical significance

The results of correlation analysis of the BMI and age with the absolute difference values of the measurements are shown in Table 5. The only significant correlation with a coefficient of 0.556 was between age and the mean difference in the measurement of the sagittal gap (*p* = 0.011).

**Table 5:** *p* values of the correlation analysis of age and BMI with the differences in the measurements

	Step axial	Step sagittal	Step coronal	Gap axial	Gap sagittal	Gap coronal
BMI	0.110	0.310	0.235	0.915	0.409	0.158
Age	0.492	0.848	0.942	0.433	0.011*	0.955

Values marked with an asterisk (*) show statistical significance

## Discussion

As acetabular fractures are articular fractures, even minor impairments of the articular surface due to displaced fracture parts can induce rapid progress of osteoarthritis and thus cause a severe disability. Following this, the scope for conservative treatments of these fractures is shallow and usually only displacements of less than 2 mm in the main weight bearing zone can be tolerated to achieve an acceptable clinical outcome [11]. So, open reduction and internal fixation is the method of choice in many cases. In open reduction, anatomical reduction is crucial for the outcome of these fractures to avoid early need for total hip arthroplasty what is associated with higher mortality and impairment [12, 13].

The possibility to intraoperatively acquire 3D data of a surgical field to evaluate reduction and implant placement is a valuable adjunct to 2D fluoroscopy as shown in several studies [5, 14, 15]. Often, these 3D scans are considered equal to CT scans regarding the parameters reduction and implant placement. Standardized technical evaluation of possible image quality of intraoperative and image quality in anatomical regions without implants has been described in several publications [16, 17].

However, the technical means of image acquisition differ between the two methods. In 3D C-arms, a set of 2D images (typically 50 to 100) is acquired during an automatized rotation around the region of interest. The 3D data is computed from these images. This limits the size of the 3D volume to a cube with an edge length of typically 12–14 cm. Additionally, due to the non-continuous data, the contrast discrimination is lower than in CT data and the data is more prone to implant artifact disturbances. CT data on the other hand is usually acquired during a continuous emittance of radiation which increases the amount of data available for image reconstruction. Thus, CT data usually has a higher contrast discrimination and is less disturbed by metal implants as there are more data available for metal artifact reduction algorithms [18, 19].

**Fig. 3:**
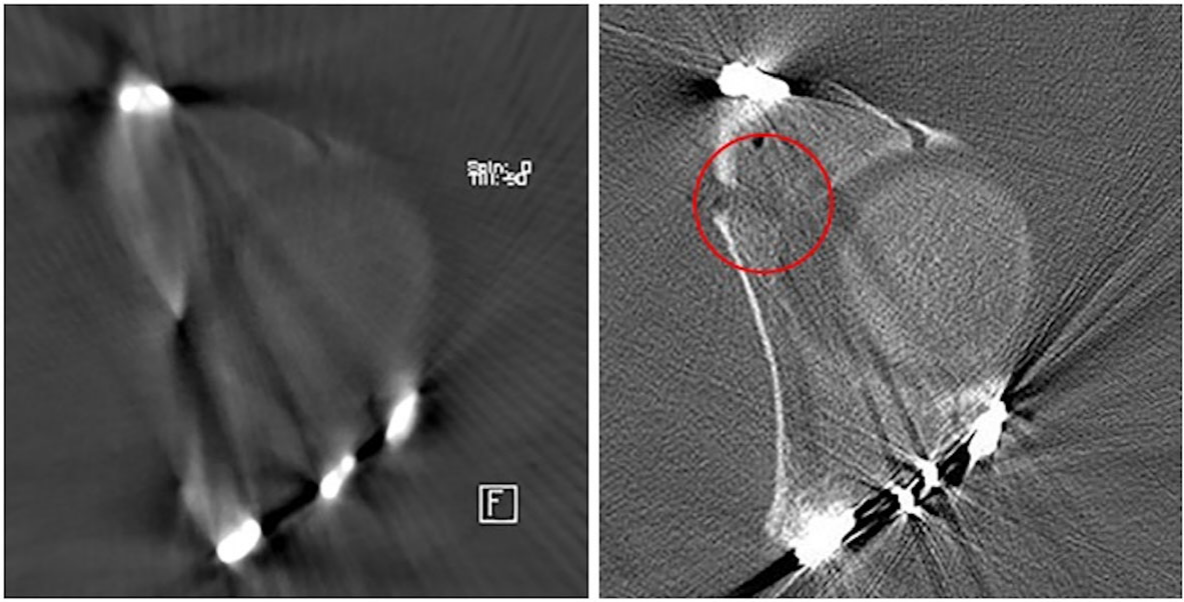
Illustration of blurring of a gap in the articular surface due to metal artifacts in the 3D scan. In the CT scan, the gap is clearly visible (red circle)

In most cases, though, the quality of 3D data acquired by 3D C-arms is sufficient to assess reduction and implant placement as demonstrated by many groups in literature that show a good assessability of the 3D data [7, 9, 10, 20–22]. However, in the special circumstance of displaced acetabular fractures with many metal implants close to the articular surface, relevant remaining steps and gaps can be missed in the intraoperative 3D imaging (see Fig. 3) what was shown in this study. This implies that the surgeon, who evaluates the imaging does not only have to assess the detectable joint line regarding reduction and the implant placement but also to judge if the 3D imaging itself is sufficient regarding the visible joint line and areas blurred by artifacts.

As shown in the evaluation of the 3D and CT data, the values for steps and gaps are rather underestimated in intraoperative 3D data. This might arise from the fact that, if large parts of the acetabular surface are not visible due to metal artifacts, possible steps and gaps are overlapped by these and thus not measured. This point emphasizes the need for proper assessment of the overall appropriateness of the intraoperative imaging as remaining relevant steps or gaps can be worse regarding the outcome than a separate revision surgery.

There are few papers that deal with the impact of the BMI on image quality. Usually, it is described that in stationary CT scanners, the patient dose is increased in obese patients [23, 24]. In intraoperative settings, the dose often cannot be increased due to power limitations. Thus, it was expected, that the BMI would have a significant impact on the assessability of the imaging. In this study, this could not be evidenced, what might be due to the rather low BMI values in our rather young collective.

Also, the correlation between the age of the patient and the assessability was only significant in the measurement values for the largest visible gap in the sagittal plane, thus not showing a strong correlation. It was assumed that age would accompany with decreased bone mineralization and thus hindered assessability in the 3D scans. There is some literature regarding the experiences in the cone beam computed tomography of the head that could show a negative correlation between age and image quality [25]. Probably, due to the small group size and rather young patients, this could not be shown.

The 3D C-arm that was used in this study represents the current clinical standard—an image intensifier based mobile C-arm. Recently, new types of 3D C-arms with flat panel detectors as well as intraoperative CT-solutions have been announced or released. These novel devices promise a significant improvement of artifact reduction and contrast resolution and have to be re-evaluated as soon as they become clinically available.

## Conclusions

In this study it was demonstrated that there are situations when steps and gaps can be missed in the intraoperative 3D imaging, especially in complex fracture situations with a high implant load close to the joint surface. In these cases, one should be aware of the potential diagnostic gap and perform a postoperative CT to ensure reduction quality and implant placement.

## Abbreviations

BMI: Body mass index
CT: Computed tomography
FFP: Fresh frozen plasma
PACS: Picture Archiving and Communication System
RBC: Red blood cells
